# Treatment of acetabular chondral lesions with microfracture technique

**DOI:** 10.1051/sicotj/2017027

**Published:** 2017-06-14

**Authors:** Claudio Mella, Alvaro Nuñez, Ignacio Villalón

**Affiliations:** 1 Departamento de Traumatología, Clínica Alemana Santiago Chile; 2 Facultad de Medicina, Universidad del Desarrollo Santiago Chile

**Keywords:** Hip, Arthroscopy, Microfractures, Chondral lesions, Indication, Surgical technique

## Abstract

*Introduction*: Acetabular cartilage lesions are frequently found during hip arthroscopy. In the hip joint they mostly occur secondary to a mechanical overload resulting from a pre-existing deformity as hip dysplasia or femoroacetabular impingement (FAI). Lesions identified during arthroscopy can vary greatly from the earliest stages to the most advanced (full-thickness lesions). These lesions occur in the acetabulum in the early stages of joint damage. Microfractures are indicated in full-thickness chondral defects. Ideally, these lesions must be focal and contained.

*Methods*: The procedure begins debriding all the unstable chondral tissue of the lesion. The edges should have a net cut towards stable and healthy cartilage. It is recommended to make as many perforations as possible using arthroscopic awls. They should be ideally 4 mm deep and must have a vertical orientation to the surface. The suggested distance between perforations is of 3–4 mm. Once the treatment of the chondral lesion with the microfractures is complete, the labrum must be repaired. The repair of the labrum transforms in most of the cases the defect in a contained lesion containing better the clot in the lesion after the microfractures have been performed. It is also important to correct the bone deformity that has caused this lesion, which mostly corresponds to a “cam” deformity.

*Conclusion*: Clinical studies confirm good short- and medium-term results in full-thickness chondral lesions treated with microfractures in the absence of osteoarthritis. However, it is difficult to determine if these results are only due to the microfractures, as this treatment is always complemented with several other factors and surgical procedures, such as labrum repair, correction of underlying bone deformity or change in postoperative activity of operated patients.

## Introduction

Acetabular cartilage lesions are frequently found during hip arthroscopy. The arthroscopic view offers an exceptional perspective to assess cartilage injuries especially in their early stages. Recent literature showed that articular cartilage damage occurs progressively up to the development of hip osteoarthritis requiring joint replacement. Numerous studies have shown that advanced cartilage damage is an indicator of a bad prognosis when performing hip arthroscopy [1, 2]. It is important to identify before surgery, patients with advanced cartilage damage who are not candidates for a hip preservation procedure (hip arthroscopy).

Several classifications exist to assess the cartilage damage in other joints, which have also been used in the hip (Outerbridge, International Cartilage Research Society, ICRS) [3]. However, the hip has special conditions that make it different from other joints. It is exceptional that these lesions occur secondary to a trauma with a normal hip anatomy. They mostly occur secondary to a mechanical overload resulting from a pre-existing deformity as hip dysplasia or femoroacetabular impingement (FAI). These pathological forces on the cartilage of the acetabulum (overload, shearing forces) occur initially in the peripheral portion of the acetabulum. Femoral cartilage damage occurs in more advanced stages of the disease as a sign of osteoarthritis of the hip.

Lesions identified during arthroscopy can vary greatly from the earliest stages to the most advanced. The progression of the chondral damage in the hip is relatively systematic. The following questions always arise, regardless of the type of lesion found during the arthroscopy: Is the injury reparable? What kind of repair is recommended? What is the prognosis and risk factor of developing osteoarthritis? To answer these questions we need a classification that allows the surgeon to guide the treatment, including prognostic factors, permitting also to compare the results of various cartilage repair techniques.

The most common classifications of chondral lesions used in the hip are the classifications of Outerbridge [3], ICRS, Konan et al. [4], Beck et al. [5] and also from Sampson [6]. In all of them the most advanced stage (Type 4) is described as a full-thickness chondral lesion with exposure of the subchondral bone. These lesions occur in the acetabulum in the early stages of joint damage. Microfractures are the suggested treatment for these types of lesions, based on the results of published experiences in other joints (especially the knee) [7]. In the hip, the treatment with microfractures in focal chondral lesions has also shown to have satisfactory results in the short-term follow-up [8–11].

Full-thickness chondral lesions of the hip have significant pathophysiological differences compared to other joints. As an example, these grade 4 lesions in the knee are often focal traumatic lesions with a generally undamaged cartilage in the rest of the joint. In the hip, these full-thickness lesions are usually the result of repetitive trauma by impingement or dysplasia. The full-thickness focal lesion is the most damaged area, but there will also be minor damages to the cartilage close to the type 4 lesions and the rest of the articular surface, especially in the acetabulum. Thus, beyond the effective treatment of the chondral lesion through microfractures, long-term results in joint preservation of the hip will also significantly depend on the quality of the rest of the cartilage as well as on an adequate correction of the underlying bone deformity.

Performing microfractures in full-thickness chondral lesions is a marrow-stimulating procedure or strategy to repair the chondral defect of the joint. The purpose is to bring undifferentiated stem cells and growth factors into the chondral defect. Penetration of subchondral tissue leads to the formation of an initial clot, which, with the advent of undifferentiated cells and growth factors, allows differentiation into chondrocytes and fibroblasts. The result will be the formation of fibrocartilage, which will cover the chondral defect.

## Indications of microfractures in chondral hip injuries

**Figure 1.**
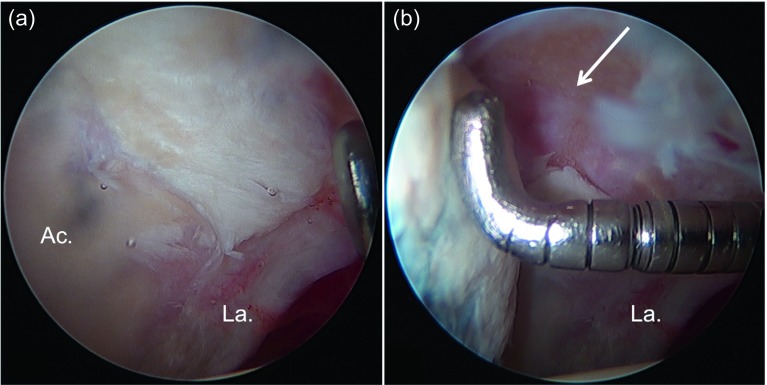
(a) Intraoperative image of hip arthroscopy of a 42-year-old male patient operated for femoroacetabular impingement (FAI). The assessment of the cartilage in the acetabulum (Ac.) shows an extensive unstable focal lesion in the anterolateral region near the acetabular labrum (La.). (b) The evaluation with a probe shows complete instability of the full-thickness chondral flap as well as exposure of the subchondral acetabulum bone (arrow).

Microfractures are indicated in full-thickness chondral defects (Outerbridge 4, ICRS 4, Konan/Haddad and Beck 4). If there is a full-thickness chondral flap with exposed subchondral bone, microfractures will also be indicated after the unstable chondral flap has been resected. Ideally, these lesions must be focal and contained (Figure 1). The problem is that in the acetabulum they usually begin at the chondrolabral junction. However, repairing the labrum in the acetabular ridge can be considered as an equivalent containment, which allows containing the clot and tissue regrowth after the microfractures have been performed. Ideally, the defect must not be larger than 4 cm^2^.

Contraindication for microfractures will be defects larger than 4 cm^2^ as well as in cases of more advanced osteoarthritis (extensive acetabular lesions, equivalent lesions of the femoral head). Age (e.g. over 60 years) is also considered a relative contraindication as well as the impossibility of undergoing an adequate rehabilitation regime [12].

## Surgical technique

**Figure 2.**
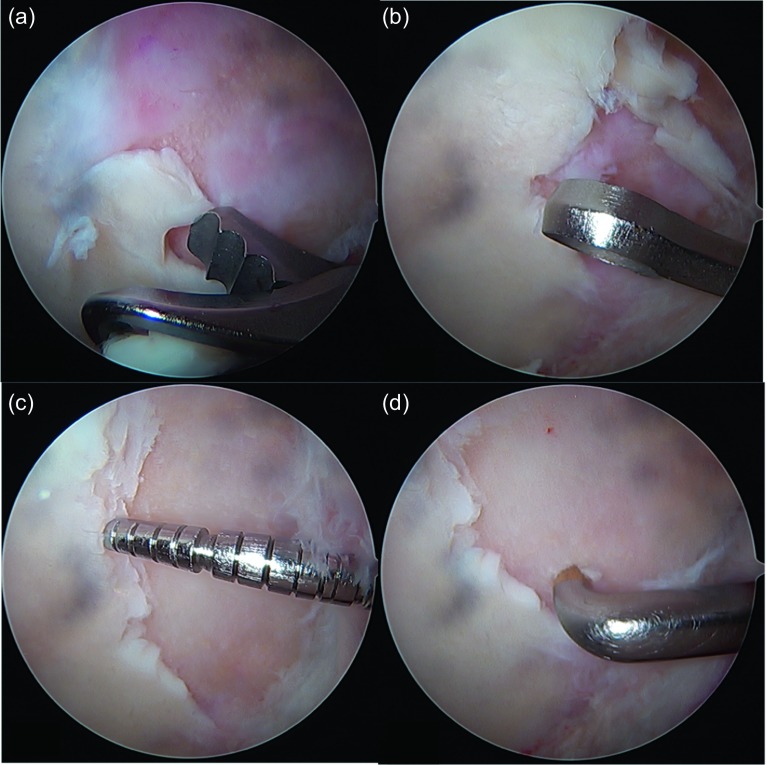
Treatment of the full-thickness focal chondral lesion: (a) Resection of the unstable chondral tissue is performed in the first instance with either shaver or tissue resection. (b) The unstable edges of the lesion are then resected with a ringed curette, leaving the edge as stable and vertical as possible; also the calcareous layer of the exposed subchondral bone is removed with the curettes. (c) After this procedure, it is recommended to palpate the edges of the lesion to confirm the stability of the remaining tissue as well as to measure the chondral defect in millimetric scale. (d) The microfractures are then made with an arthroscopic awl beginning at the vertex of the lesion. For this purpose, there are awls of different angulation to make the perforations as vertical as possible to the lesion.

**Figure 3.**
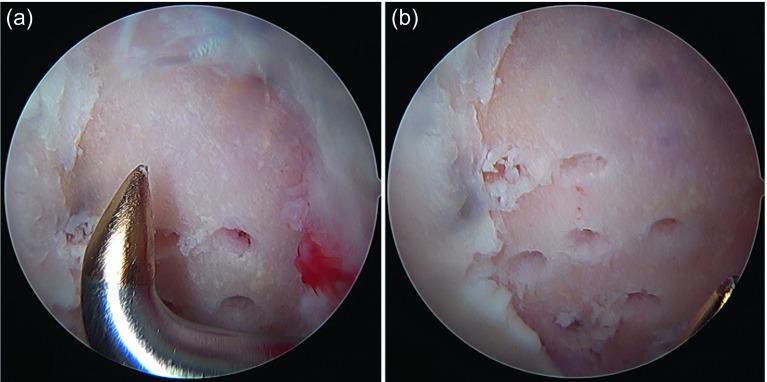
(a) The microfractures are performed systematically with an ideal depth of 4 mm and a distance of 3–4 mm between the perforations. (b) The entire surface should be covered with perforations including microfractures close to the edge of the lesion.

**Figure 4.**
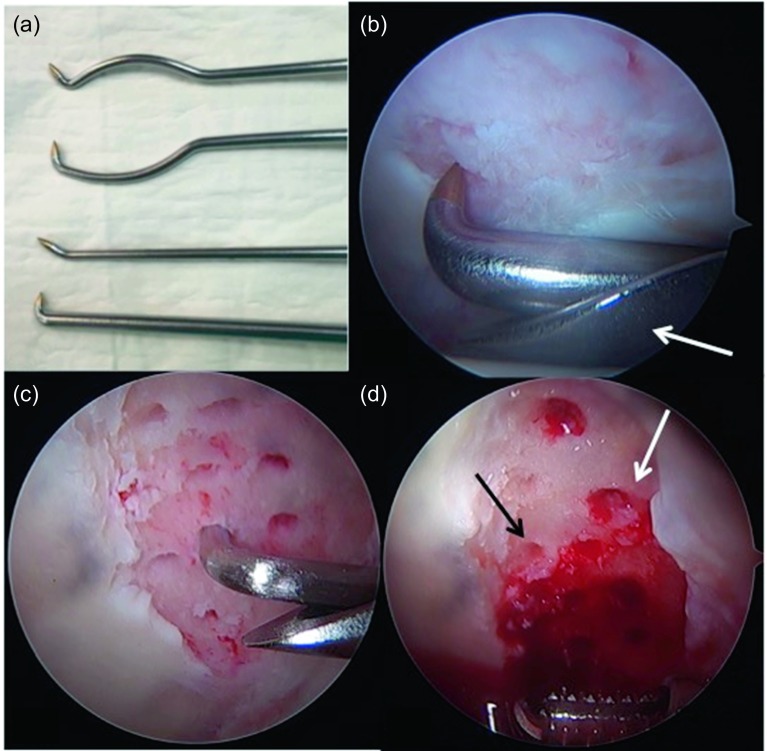
(a) There are different designs of arthroscopic awls with diverse angles to compensate the mechanical difficulty performing microfractures in the reduced space of the hip joint. (b) When making the impact with the mallet on the “arthroscopic awl” there is a risk of sliding forward creating grooves instead of perforations. To prevent this, it is beneficial to use the supplementary support on a hemi-cannula (arrow). (c) This protects the cartilage from the femoral head and allows pressuring against the acetabular surface, achieving a greater penetration of the awl at the moment of impact preventing the creation of grooves on the surface. (d) Once the microfractures have been completed, articular irrigation can be suspended to verify adequate bleeding from the perforations (white arrow) and to further deepen the perforations where there is still no bleeding (black arrow).

A big advantage of microfractures in focal chondral lesions is that it is a reproducible and inexpensive surgical technique with a low rate of morbidity and complications. It has already proven long-term clinical results (in the knee). The procedure begins debriding all the unstable chondral tissue of the lesion (with resection clamps or shaver) (Figure 2a). The edges should have a net cut towards stable and healthy cartilage; this can be done with ringed curettes (Figure 2b). When the lesion has been delimited (Figure 2c) and the unstable tissue has been resected, the calcified layer of subchondral bone must also be removed using the ringed curettes. It is recommended to make as many perforations as possible using arthroscopic awls [13]. They should be ideally 4 mm deep and must have a vertical orientation to the surface (Figure 2d). The suggested distance between perforations is of 3–4 mm (Figure 3). After the procedure, the bleeding from the perforations by suspending the joint perfusion can be observed (Figure 4) with the option to further deepen the perforations where there is still no bleeding. When finishing the perforations, the residual unstable tissue and residues must be removed with shaver or curettes.

To perform these micro perforations in the hip presents special technical difficulties compared to the knee. The joint space is very small, which makes access difficult with the usual instruments and poses the risk of injuring the cartilage adjacent to the lesion or the femoral head. There are different designs of arthroscopic awls (Figure 4a); different companies have developed awls with diverse angles to compensate this mechanical difficulty. Motorized drills have also been developed to achieve optimal perforations with adequate depth and spacing. Another problem is the angle from which the injury is accessed with the drill or arthroscopic awls that will be oblique to the surface of the lesion. On the other hand, the direction of the force of impact will be horizontal and not vertical to the lesion, with the risk of sliding with the arthroscopic awl, making grooves in the surface and not the perforations with the necessary depth. To prevent this, it is beneficial to use the supplementary support on a hemi-cannula (Figures 4b and 4c). This protects the cartilage from the femoral head and allows pressuring against the acetabular surface, achieving a greater penetration of the awl at the moment of impact preventing the creation of grooves on the surface. When using the mallet, the awl must be firmly pressed towards the bone and with some traction to prevent the awl from slipping in.

**Figure 5.**
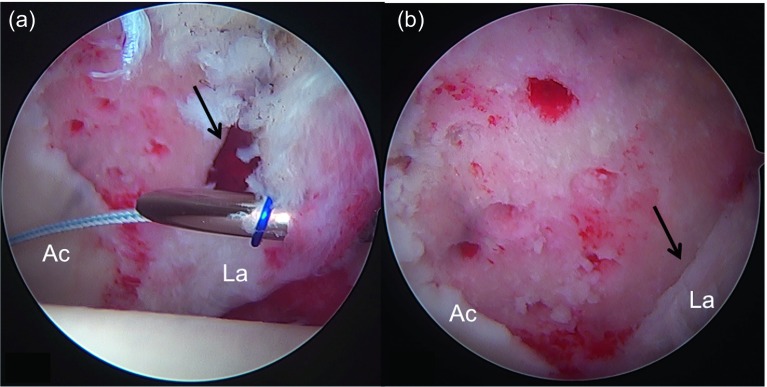
Once the microfractures are completed in the acetabulum (Ac) and the acetabular resection osteoplasty is performed, the acetabular labrum (La) must be repaired. This is important since it is the only option to transform this open lesion at the chondrolabral rim (a, arrow) into a contained defect. In this case, a translabral suture (a) was used to fix the labrum to the acetabular ridge. Once the suture was completed, a suitable apposition of the labrum on the edge of the lesion can be observed (b, arrow) sealing the limit of the chondral lesion. This allows a better containment of the clot generated in the area of the chondral lesion treated by the microfractures.

Once the treatment of the chondral lesion with the microfractures is complete, the labrum must be repaired. This is of paramount importance in these full-thickness lesions that usually started at the acetabular rim (Figure 5a). The repair of the labrum transforms the defect in a contained lesion containing better the clot in the lesion after the microfractures have been performed (Figure 5b). Important is also to correct the bone deformity that has caused this lesion, which mostly corresponds to a “cam” deformity. Correction of this deformity through an arthroscopic femoroplasty will be essential to reduce the impact in the repaired area and, if possible, to prevent progressive joint deterioration.

After the surgery all patients started physical therapy on the same day to initiate range of motion using a continuous passive motion machine for three hours. On the first postoperative day they started using the stationary bicycle three times a day for 20 min. After performing microfractures we recommend the use of partial weight bearing for six weeks before full weight bearing is allowed.

## Complementary biological treatments

Although microfracture treatment is clearly suggested for the management of full-thickness chondral lesions, there are still deficiencies of neoformed tissue in the lesion. On the one hand, the initial formed clot is highly unstable and with a low amount of pluripotent cells. On the other hand, the result will always be the formation of fibrocartilage and not of hyaline cartilage, which would be the desired tissue for this type of lesion. However, various complementary biological treatments have been suggested, but they will always be complementary to the microfractures with the aforementioned technique [14, 15]. Options for complementary biological treatments include the use of growth factors (Plasma Rich Protein, PRP), collagen membranes, stem cells, chondrocyte grafts or, in case of open surgery, osteochondral grafts. All these complementary options are surgically more demanding, especially the stable fixation of the respective membranes in the area of the microfractures. They also have additional surgery costs. Recent publications confirmed the formation of a more stable tissue, with higher cellularity and good short-term clinical outcomes but there are insufficient long-term clinical results supporting the routine use of some of these surgical techniques [14–16].

## Outcomes

One of the first publications about short-term clinical results in patients treated with microfractures for grade 4 chondral lesions of the hip was by Philippon et al. [8]. In 2008, he published a series of nine patients who underwent revision arthroscopy after previous arthroscopic treatment with microfractures for a full-thickness chondral lesion. He describes filling an average of 91% with a tissue described as stable. However, no clinical results of these series were published.

Publications that are more recent confirm good short-term clinical results. In 2009, Byrd and Jones [9] published a series of micro perforations in 58 patients with grade 4 lesions with good functional results and an increase of 20 points (rise from 65 preop. to 85 postoperative) in the Modified Harris Hip Score (mHHS). In 2012, Karthikeyan et al. [17] published a series of 20 patients who underwent microfractures in acetabular chondral defects. Revision arthroscopy was performed in all of them (17 ± 11 months of follow-up). An average filling of the chondral defect of 93% (±17%) was found, defining the cartilage as macroscopically stable. The results in functional scores showed an increase of the non-arthritic hip score (NAHS) from 54.5 to 78 in an average period of 21 months. In 2014, Domb et al. published a series of 30 patients with femoroacetabular impingement (FAI) and grade 4 chondral lesions [18]. In all of the patients the FAI was corrected, the full-thickness chondral lesions were treated with microfractures. The clinical assessment was performed with a minimum period of two years of follow-up, considering several functional scores (mHHS, NAHS, HOS-ADL). All measured functional scores had significant improvements; however, there was no comparative group in this study. In 2015, Domb et al. published a new series of patients with a femoroacetabular impingement and chondral lesions treated by hip arthroscopy [19]. Two comparative groups with and without microfractures were defined. The aforementioned functional scores were assessed after a minimum follow-up period of two years. Although all patients showed a significant improvement in all measured scores, there was no significant difference in the two studied groups.

In 2012, McDonald et al. also published a comparative series of elite athletes treated with and without microfractures during the arthroscopic treatment of femoroacetabular impingement [20]. The two study groups presented no significant differences when resuming their sports activities.

In 2016, Marquez-Lara et al. published a systematic review of indications, outcomes and postoperative-treatment rehabilitation protocols using microfractures in patients with chondral lesions secondary to femoroacetabular impingement [21]. Twelve studies (11 out of 12 studies) showed good post-microfracture results in 267 patients (except one publication of a case report with one clinical case). As an indication for microfractures, the vast majority considered full-thickness focal chondral lesions. Most publications also recommend some degree of weight-bearing protection in postoperative rehabilitation, nevertheless rehabilitation protocols vary significantly.

In 2015 Fontana and de Girolamo published a study comparing the clinical results after five-year follow-up of microfracture (MFx) with a technique of enhanced microfracture autologous matrix-induced chondrogenesis (AMIC) for acetabular chondral lesions grades 3 and 4 [22]. The outcome in both groups was significantly improved at six months and one year postoperatively. During the subsequent four years the outcome in the MFx group deteriorated slowly, whereas that in the AMIC group remained stable. They conclude that at the short term clinical outcomes improve in both Mfx and AMIC groups. However, the AMIC group had better and more durable improvement.

In summary, studies confirm good short- and medium-term results in full-thickness chondral lesions treated with microfracture, in the absence of osteoarthritis. However, it is difficult to determine if these results are only due to the microfractures, as this treatment is always related to several other factors and surgical procedures, such as labrum repair, correction of underlying bone deformity or change in postoperative activity (Impact sports reduction) of operated patients.

## Final thoughts

Acetabular chondral lesions are considered a relevant prognostic factor when performing a hip arthroscopy. Their correct classification allows choosing the optimal treatment of the chondral injury. There are several options available to treat these lesions through hip arthroscopy, which combine microfracture therapies with several complementary biological treatments. However, beyond the treatment effectiveness of the focal chondral lesion in the acetabulum, the hip presents other related factors, which will significantly influence the long-term results. Unlike the knee, in which chondral injuries occur often due to traumatic injuries but leave a healthy remaining cartilage, the remaining cartilage in the hip is often damaged to a varied extent (thinning, cracking, etc.), in addition to the focal lesion, for which there are currently no arthroscopic treatment options. On the other hand, proper correction of the underlying bone deformity (impingement, hip dysplasia) will be essential in the long-term prognosis of the hip treated by hip arthroscopy.

## Conflict of interest

The authors declare no conflict of interest in relation to this paper.
